# Transformational Applications of Human Cardiac Organoids in Cardiovascular Diseases

**DOI:** 10.3389/fcell.2022.936084

**Published:** 2022-06-09

**Authors:** Wanling Xuan, Srinivas M. Tipparaju, Muhammad Ashraf

**Affiliations:** ^1^ Department of Pharmaceutical Sciences, USF Health Taneja College of Pharmacy, University of South Florida, Tampa, FL, United States; ^2^ Vascular Biology Center, Medical College of Georgia, Augusta University, Augusta, GA, United States

**Keywords:** organoids, heart, tissue culture, iPSC, stem cells

## Abstract

Organoid technology has significantly advanced in recent years and revolutionized the field for generation of organs using *in vitro* systems (a.k.a “organs in a dish”). The use of pluripotent stem cells or tissue derived cells for generating a 3-dimensional culture system to recapitulate the architecture and function of the organ is central in achieving and improving organoid systems. Unlike most organs in the body, very little progress has been made in cardiac organoid due to its structural complexity and vascularization. In this review, we will discuss the current applications of human cardiac organoids for cardiac disease modeling, drug discovery, drug cardiotoxicity testing, and clinical applications.

## 1 Introduction

The organoids are described as cells that are grown in a distinct 3-dimensional environment *in vitro*, creating mini-clusters that undergo self-organization and division into functional organ in the dish. (Corrò et al., 2020). Recent advances in the organoid technology have revolutionized the *in vitro* culture tools for biomedical research by creating powerful three-dimensional (3D) models to recapitulate the cellular heterogeneity, structure, and functions of the primary tissues. Organoids have been used in disease modeling and drug discovery with a wide range including brain (Lancaster et al., 2013), kidney (Takasato et al., 2015), liver (Hu et al., 2018), intestine (Fujii et al., 2018). However, relatively less progress has been made in cardiac organoids due to structural complexity and vascularization. Currently, there are two major approaches for generation of cardiac organoids including direct assembly of either primary cardiac cells or pluripotent stem cells (PSCs) derived cardiac cells and self-organization by PSCs. Cell culture techniques involving primary and immortalized cells are mainstream approaches which are used to recapitulate some facets of the human disease for investigating disease mechanisms and pharmacological treatments. However, the lack of direct relevance of organoids to human diseases due to cellular complexity of the tissues and organs has posed significant limitations and decreased their translational potential and clinical relevance. Despite these limitations, human cardiac cells derived organoids hold great potential and promise in modeling human cardiovascular diseases for mechanistic studies, drug development and precision medicine. This progress has been made possible due to the invention of induced pluripotent stem cells, advanced imaging, molecular techniques, and 3D organoid technology (Kim et al., 2022).

In this review, we present the current applications of human cardiac organoids in cardiac diseases modeling, including congenital heart diseases, myocardial infarction, cardiomyopathy, and heart failure. In addition, we will also discuss the application of organoid technology in drug screening for cardiovascular disease therapy and drug cardiotoxicity testing. Finally, we will explore the clinical applications of cardiac organoids, limitations, and future directions.

## 2 Application of Cardiac Organoids

### 2.1 Heart Diseases Modeling

#### 2.1.1 Congenital Heart Disease

Congenital heart defect (CHD) is among the most common types of birth defects and the major complications are left ventricular non-compaction cardiomyopathy and heart failure. However, the molecular mechanisms and origin of these disorders remain poorly understood. Despite the use of transgenic mouse models, the need for human relevant disease models remains in high demand for research and translational application, with major emphasis on models designed to recapitulate post-natal and developmental regulation of heart in early stages. Human pluripotent stem cells (hPSCs), including embryonic stem cells (hESCs) and induced PSCs (hiPSCs), can differentiate into any cell type in the body. hPSCs can form the 3D tissue resembling embryo-like tissue patterns *in vitro* by manipulating cardiac developmental program, which enable researchers to study human cardiac development and cardiac defects.

The previous research work points to major pathways and mechanisms using *in vitro* models that allow to identify the similarities and molecular features that complement findings obtained *in vivo* towards human disease. Drakhlis et al. combined directed cardiac differentiation by Wnt pathway modulation and encapsulation of hPSCs in matrigel to generate self-organizing cardiac organoids which resembled early embryonic heart anlagen (Drakhlis et al., 2021). Nkx2.5 is an important cardiac transcription factor for heart development and its mutations lead to human structural cardiac defects (McElhinney et al., 2003; Zhang et al., 2014). In addition, Drakhlis et al. studied genetic defects using Nkx2.5 knock out hESCs (HES3-Nkx2.5-KO) derived cardiac organoids and reported that Nkx2.5 deletion showed a phenotype reminiscent of cardiac malformations previously observed in transgenic mice. These findings highlight that cardiac organoids likely mimic properties of human heart *in vitro*. However, cardiac organoids are far away from structural and cellular complexity of the heart. Similarly, Lewis-Israeli et al. used three sequential Wnt modulation steps (activation/inhibition/activation) for cardiac mesoderm induction and epicardial induction at specific time points on embryoid bodies (EB) with suspension culture to generate cardiac organoids. Transcriptomic analysis reveals that organoids closely model human fetal cardiac development and produce multiple cardiac-specific cell lineages with a composition of cardiomyocytes, cardiac fibroblasts, endocardial cells, and endothelial cells. Importantly, these organoids displayed well-defined sarcomeres surrounded by mitochondria, gap junctions and the presence of tubular structures reminiscent of T-tubules. Robust beating and normal electrophysiological activity with well-defined action potential waves reminiscent of QRS complexes, T and P waves and regular action potentials were also detected using a multi-electrode array. This study strongly supports that heart organoids are functional and will likely serve as good candidates for disease modeling.

Furthermore, the combination of growth factors, bone morphogenetic protein 4 and activin A improved heart organoid chamber formation and vascularization (Lewis-Israeli et al., 2021a; Lewis-Israeli et al., 2021b). Pregestational diabetes (PGD) which starts before pregnancy and present during at least the 1st trimester of fetal development affects nearly 2% of all pregnancies (Pauliks, 2015), and increases the risk of fetal congenital heart disease. It is also known to cause fetal hypertrophic cardiomyopathy and impairs cardiac function in infants (Pauliks, 2015). Lewis-Israeli et al. utilized the heart organoid platform to model the effects of PGD on the developing heart. This platform recreates complex metabolic disorders with decreased oxygen consumption and increased glycolysis in pregestational diabetes-like conditions (Lewis-Israeli et al., 2021b). Overall, these studies provide the basic constituents for developing cardiac organoids derived from PSCs which might serve as good models for studying molecular pathology of CHD in humans.

#### 2.1.2 Ischemic Heart Disease

Ischemic heart disease (IHD) is a leading cause of death worldwide, which manifests clinically as myocardial infarction (MI) (i.e., heart attack) and ischemic cardiomyopathy (Shao et al., 2020). Heart attacks are a common cause of heart failure with a 40% 5-year mortality (Lauridsen et al., 2021). During the past decades, clinical trials show limited success in heart failure for translation using information from animal models to human studies. Human cardiac organoids showed a great translational potential to bridge and connect basic research findings using animal models of heart failure and transition towards human studies.

Newborn rodent hearts possess an endogenous regenerative capacity following injuries including ventricular resection, cryoinjury, and coronary artery ligation (Lam and Sadek, 2018; Shen et al., 2021); however, such regenerative capacity rapidly diminishes during the first 7 days after birth in mice. Advances have been made to understand the underlying mechanism for such loss of mammalian cardiac regenerative potential and to identify the potential approaches for boosting regenerative capacity in adulthood upon injury (Hirose et al., 2019). Voges et al. generated cardiac organoids by fabrication of hESCs derived cardiac cells in the mold and showed that following cryoinjury the human cardiac organoids exhibited an endogenous regenerative response with complete functional recovery. Importantly, this model provides a unique opportunity to explore the molecular and cellular mechanisms governing regeneration of immature human heart tissue (Voges et al., 2017). The bioengineered heart organoids provide a great potential, however there are currently limitations since organoids do not display initial ECM accumulation typical for the early stages of both regeneration and fibrosis (Voges et al., 2017; Hofbauer et al., 2021). Hofbauer et al. established hPSCs derived self-organizing “cardioids” that recapitulate chamber-like morphogenesis in the absence of non-cardiac tissues (Hofbauer et al., 2021). These cardioids contained all three major cardiac lineages, solely relying on developmental mechanisms without external ECM scaffolds and produced a more physiological response upon cryoinjury. In trilineage “cardioids”, a rapid recruitment of either endothelial cells or epicardial associated fibroblast-like cells was observed after cryoinjury, suggesting they could mimic an important early aspect of regenerative and fibrotic response (Hofbauer et al., 2021). Richards et al. utilized oxygen diffusion together with chronic adrenergic stimulation to create a gradient of “apoptotic center-dysfunctional interior functional edge” in human cardiac organoids generated by fabrication of 50% hiPSCs derived cardiomyocytes and 50% non-myocytes. This setting recapitulated the “infarct-border-remote zones” of infarct hearts. In addition, the composition of multiple cell types facilitated to mimic organotypic myocardial response to infarction in cardiac organoids including fibrosis and pathological calcium-handling. They also demonstrated that organoid based myocardial infarction model recapitulates hallmarks of myocardial infarction (pathological metabolic shifts, fibrosis, and calcium handling) (Richards et al., 2020).

#### 2.1.3 Non-ischemic Cardiomyopathy

Non-ischemic cardiomyopathy is a major underlying cause of heart failure (Seferovic et al., 2019). The pathophysiology of non-ischemic cardiomyopathy is multifactorial and includes genes and environment interactions along with other comorbidities (Wu, 2007; Shanbhag et al., 2019; Lombardi and Chen, 2020). Tiburcy et al. used human cardiac organoids generated with PSCs derived cardiomyocytes, fibroblasts, and ECM to model heart failures with isoprenaline and norepinephrine stimulation. Cardiomyocyte hypertrophy was developed in response to catecholamine stimulation, in which the cardiac organoids showed a loss of a positive force-frequency-response, and adrenergic signal desensitization (Tiburcy et al., 2017).

iPSCs derived from patient tissue and utilized for mutagenic studies allow for the development of cardiac organoids which are physiologically relevant to model familial cardiomyopathy under *in vitro* settings. Recently, Yang et al. generated cardiac constructs by direct assembly of hiPSCs derived cardiomyocytes and human marrow stromal cells from myosin heavy-chain 7 mutation (E848G) patients. These cardiac organoids carrying E848G mutation exhibited significantly less alignment, significant reduction of systolic function and minimal impact on diastolic function (Yang et al., 2018). Similarly, Buono et al. directly assembled iPSCs derived cardiomyocytes from hypertrophic cardiomyopathy (HCM)-patient carrying an MYH7 mutation and human cardiac endothelial cells and cardiac fibroblasts at a physiological cell ratio of 3:5:2 to construct cardiac organoids which displayed arrhythmia phenotype (Filippo Buono et al., 2020). Long et al. generated engineered heart muscle using Duchenne muscular dystrophy (DMD) patient hiPSCs derived cardiomyocytes and human fibroblasts to model DMD cardiomyopathy. They also generated the heart muscle using cardiomyocytes with correction of DMD mutation by CRISPR/Cas9 technology. After correction of DMD mutation, dystrophin expression was restored, and the heart tissue showed improvement of mechanical contraction (Long et al., 2018). This study provides an insight for genetic cardiomyopathy modeling and functional effects of gene editing in human cardiac tissue level. Overall, these studies supported the approach that human cardiac organoid is useful for modeling non-ischemic cardiomyopathy with variety of pathogenesis causes.

### 2.2 Drug Development for Cardiovascular Diseases

Majority of the drugs that are approved in the pre-clinical stages of drug development fail at the clinical trial level since most of the initial testing and target validation are conducted using 2D cell culture or small animal models (Mokry et al., 2015). In recent years, hiPSCs-based disease models represent a useful and powerful tool for drug discovery. The new advances and opportunities in the field allow for drug screening using patient-specific iPSCs and differentiation of disease affected cells. In this respect, iPSCs-cardiomyocytes can be used for personalized drug screening and clinically relevant diseases models. In fact, successful pre-clinical application of iPSCs-derived cardiomyocytes for drug screening assays has been demonstrated recently, to assess the proarrhythmic risk of novel cardio therapeutics (Kussauer et al., 2019). However, despite great advantages of iPSCs for drug development, one hurdle is a lack of functional maturation of iPSCs-derived cardiomyocytes in 2D culture. In addition, the biological architecture and intercellular interactions contribute to disease pathogenesis. Therefore, organoid-based technologies offer a greater physiological relevance relative to their 2D counterparts.

Mills et al. performed drug screening to identify pro-proliferative compounds using bioengineered human cardiac organoids (Mills et al., 2019) using 2D iPSCs-derived cardiomyocytes and then in immature proliferative cardiac organoids, followed by validation in mature, cell-cycle-arrested cardiac organoids. Furthermore, under conditions recapitulating postnatal metabolic environment, maturation of human cardiac organoids can be further enhanced, which is evidenced by a metabolic switch from glycolysis to fatty acid oxidation, expression of adult sarcomere isoforms, t-tubules, adult-like electrophysiological properties, extracellular matrix remodeling, and cardiomyocyte cell-cycle arrest. The maturity of cardiac organoids provides detailed insight for pro-regenerative drug development. In combination with proteomics analysis, this study identified two pro-proliferative compounds without functional side effects and their underlying biological mechanisms.

With the emergence of SARS-Cov-2 infections, epidemiology studies show high morbidity and mortality rates in patients with pre-existing cardiovascular disease which lead to cardiac injury, dysfunction or death (Italia et al., 2021). Mills et al. also utilized the mature human cardiac organoids for drug screening in cardiac dysfunction with SARS-Cov-2 infection setting (Mills et al., 2021). They used a cocktail of inflammatory cytokines to mimic SARS-Cov-2 infection. In response to the “cytokine storm,” those cardiac organoids exhibited diastolic dysfunction with similar transcriptional profiling as hearts of SARS-Cov-2 infected K18-hACE2 mice. Subsequently, a targeted drug screen identified bromodomain and extra-terminal family (BET) inhibition is an attractive therapeutic candidate. They also validated that one of the BET inhibitor-INCB054329 prevents cardiac dysfunction in a mouse lipopolysaccharide-induced cytokine storm model and in cardiac organoids in response to COVID-19 patient serum. The study further identified two BD2-selective BET inhibitors RXV-2157 and apabetalone which were effective against COVID-19 damage (Mills et al., 2021). Overall, these studies indicated that compared with traditional 2D culture formats, human cardiac organoid screening platforms are directly relevant towards the drug development and discovery.

### 2.3 Cardiotoxicity

Cardiotoxicity is a major concern for delivery, development of systemic utilization of drugs and investigational approaches, and during different stages of approval process. Specifically, the ability to detect drug-induced exacerbation of cardiotoxicity is an unmet need for all drug development to address safety concerns for patients with pre-existing cardiovascular conditions. Impactful areas of focus include cardio-oncology for cancer patients with pre-existing cardiovascular diseases or related risk factors who are at a greater risk of cardiac complications from use of anti-cancer therapies (Curigliano et al., 2020). A previous study showed that anti-cancer drugs can cause life-threatening ventricular arrhythmias with prolonged QT and torsades de pointes (TdP) (Duan et al., 2018). Efforts have been made to assess the drug toxicity using hiPSCs derived cardiomyocytes. However, the lack of interaction between cardiomyocytes and non-myocytes may not fully represent the effects of testing drugs on cardiac structure and function.

Compared to 2D monolayer cultures, human cardiac organoids allowed for recapitulation of 3D tissue-level responses. Using 3D vascularized cardiac tissue consisting of hiPSCs derived cardiomyocytes and fibroblasts, Tadano et al. successfully detected drug-induced modulation in repolarization and contractility (Tadano et al., 2021). Archer et al. generated 3D cardiac microtissues with three major cardiac cells including cardiomyocytes, endothelial cell, and fibroblasts to detect drug induced changes in cardiac morphology. Effects of 15 FDA approved structural cardiotoxins and 14 FDA approved non-structural cardiotoxins on structure of these 3D cardiac microtissues including mitochondrial membrane potential, endoplasmic reticulum integrity and cellular viability were successfully evaluated. This study demonstrated that the 3D cardiac microtissues can be used as a high-throughput experimental model to detect cardiotoxicity in cardiac structure and to provide insights into the phenotypic mechanisms of this liability (Archer et al., 2018). Unlike the direct assembled cardiac organoids, self-organized organoids can be used for studying drug-induced cardiac developmental toxicity by quantifying the drug effects based on cardiac differentiation, contractile, and 3D tissue morphology (Hoang et al., 2021). Thus, this platform can be adopted for pharmaceutical development and fetal safety assessments. Additionally, the cardiac organoid models can be used for mimicking the pre-existing cardiovascular conditions in patients for studying enhanced doxorubicin induced cardiotoxicity (Voges et al., 2017). In summary, these studies support the use of human cardiac organoids for investigating drug-induced cardiotoxicity in humans with a high accuracy during the early stages of drug development.

### 2.4 Clinical Application of Cardiac Organoids

The recent advent of iPSC has accelerated the progress on organoids production for transplantation in target organs. The transplantation of tissue-specific organoids leads to improvement in tissue function. Transplants have been tested in mouse models for tissue engraftment, biocompatibility, and functionality. In 2014, Watson et al. generated human intestinal organoids produced *in vitro* from hiPSCs for engraftment *in vivo*. The transplanted organoids showed successful engraftment and presence of enterocytes, enteroendocrine cells, paneth cells, and goblet cells and reepithelization of damaged ileal mucosa (Watson et al., 2014). Subsequently, retinal, kidney, liver, pancreas and brain organoids have been transplanted successfully (Xu et al., 2018). Varzideh et al. developed the first hiPSCs-derived cardiac organoids for transplantation. After 24 h of organoid formation, the presence of three different cell types was observed, cardiac progenitor cells (CPC), MSCs, and endothelial cells (Varzideh et al., 2019). These cells started to self-organize into 3D organoids after 72 h, and after 1-week, cardiac organoids presented a homogeneous beating with mature cardiomyocytes. More recently, Sawa group in Japan successfully implanted allogeneic hiPSCs-CM patches in a patient with ischemic cardiomyopathy. These patches were comprised of cardiomyocytes, a component of cardiac organoids. The changes in the wall motion in the transplanted site were recovered after 6 months of transplantation. Additional ongoing studies will further show their therapeutic efficacy and safety in patients (Miyagawa et al., 2022).

## 3 Limitation and Perspectives

**FIGURE 1:**
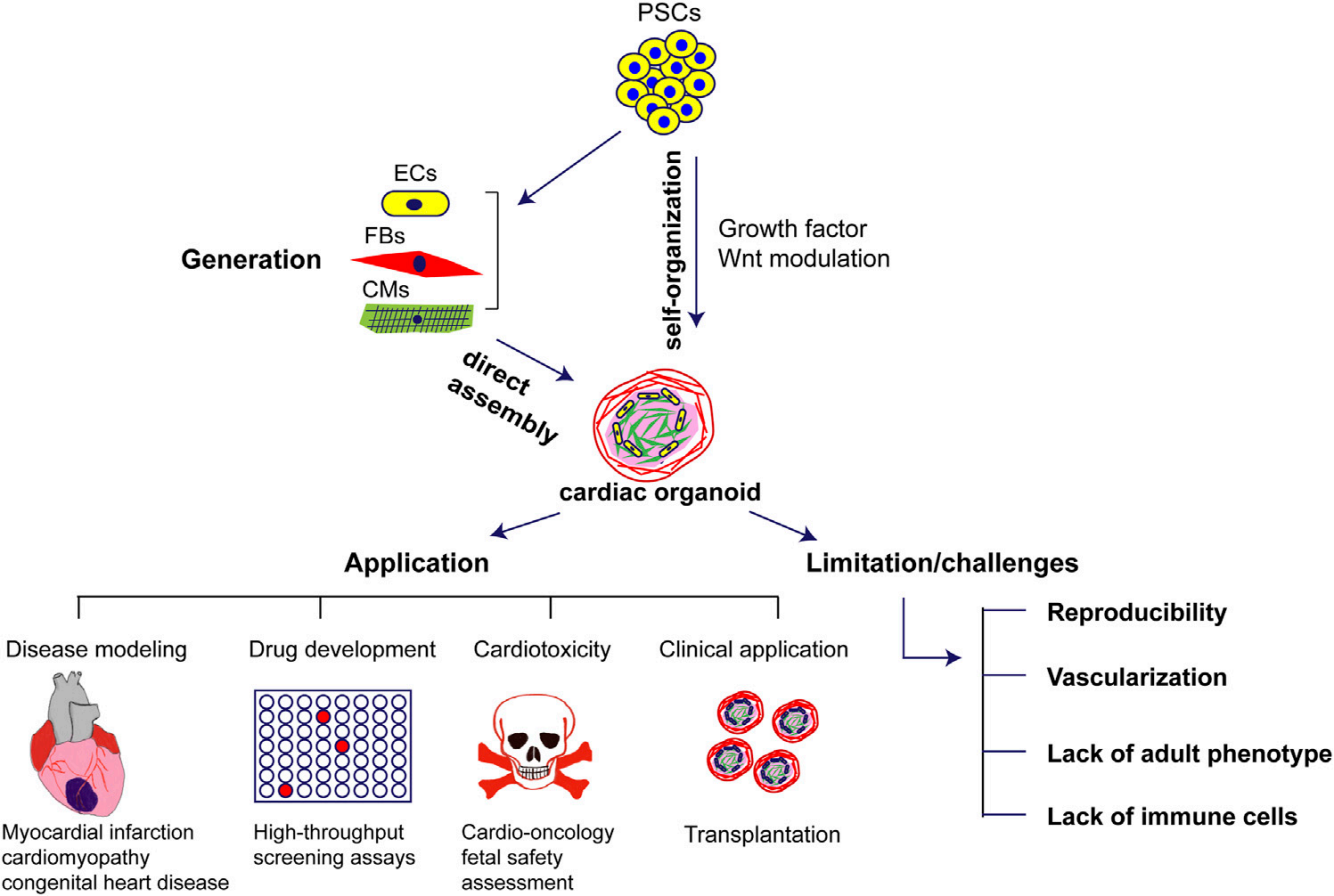
Generation, application and challenges of human cardiac organoids in cardiovascular diseases. PSCs: pluripotent stem cells; ECs: endothelial cells; FBs: fibroblasts; CMs: cardiomyocytes.

Recent advances in the generation of human cardiac organoids helped to bridge the gap between animal models of disease and drug discovery (Figure 1). There are still numerous challenges namely reproducibility, vascularization, lack of adult phenotype that need to be addressed to practically integrate cardiac organoids into preclinical studies of drug development. In combination with hiPSCs technology, reprogramming from patient specific somatic cells into hiPSCs offers not only the possibility of differentiating all cell types involved in the generation of cardiac organoids but also offers new perspectives for personalized drug screening to achieve optimal therapeutic applications and mechanistic insights. However, there are a few limitations for the current cardiac organoids and tissue engineering technology which does not enable the organoids to fully recapitulate the human heart. Overcoming the limitations of current approaches of developmental modeling by refining differentiation protocols for diverse cell types in an organoid and prolonging the viability of the organoids over time will open the field to a wide range of applications beyond fetal heart development and disease discovery. Furthermore, the current cardiac organoids mainly consist of cardiac cells with cardiomyocytes, endothelial cells, and fibroblasts but the lack of immune cells fails to address the immunological responses in cardiac inflammatory models. In this regard, vaccination with mRNA vaccines against coronavirus disease 2019 has been associated with a risk of developing myocarditis and pericarditis and the current models of organoids fail to address the pathophysiology of this disease. The ongoing rapid pace of research on cardiac organoids will likely overcome these limitations and facilitate their use for cardiac development and disease studies as well as clinical applications.

## References

[B1] Archer C. R. Sargeant R. Basak J. Pilling J. Barnes J. R. Pointon A. (2018). Characterization and Validation of a Human 3D Cardiac Microtissue for the Assessment of Changes in Cardiac Pathology. Sci. Rep. 8 (1), 10160. 10.1038/s41598-018-28393-y 29976997 PMC6033897

[B2] Corrò C. Novellasdemunt L. Li V. S. W. (2020). A Brief History of Organoids. Am. J. Physiol. Cell Physiol. 319 (1), C151–C165. 10.1152/ajpcell.00120.2020 32459504 PMC7468890

[B3] Curigliano G. Lenihan D. Fradley M. Ganatra S. Barac A. Blaes A. (2020). Management of Cardiac Disease in Cancer Patients throughout Oncological Treatment: ESMO Consensus Recommendations. Ann. Oncol. 31 (2), 171–190. 10.1016/j.annonc.2019.10.023 31959335 PMC8019325

[B4] Drakhlis L. Biswanath S. Farr C.-M. Lupanow V. Teske J. Ritzenhoff K. (2021). Human Heart-Forming Organoids Recapitulate Early Heart and Foregut Development. Nat. Biotechnol. 39 (6), 737–746. 10.1038/s41587-021-00815-9 33558697 PMC8192303

[B5] Duan J. Tao J. Zhai M. Li C. Zhou N. Lv J. (2018). Anticancer drugs-related QTc Prolongation, Torsade De Pointes and Sudden Death: Current Evidence and Future Research Perspectives. Oncotarget 9 (39), 25738–25749. 10.18632/oncotarget.25008 29876021 PMC5986642

[B6] Filippo Buono M. von Boehmer L. Strang J. Hoerstrup S. P. Emmert M. Y. Nugraha B. (2020). Human Cardiac Organoids for Modeling Genetic Cardiomyopathy. Cells 9 (7), 1733. 10.3390/cells9071733 PMC7409052 32698471

[B7] Fujii M. Matano M. Toshimitsu K. Takano A. Mikami Y. Nishikori S. (2018). Human Intestinal Organoids Maintain Self-Renewal Capacity and Cellular Diversity in Niche-Inspired Culture Condition. Cell Stem Cell 23 (6), 787–793. 10.1016/j.stem.2018.11.016 30526881

[B8] Hirose K. Payumo A. Y. Cutie S. Hoang A. Zhang H. Guyot R. (2019). Evidence for Hormonal Control of Heart Regenerative Capacity during Endothermy Acquisition. Science 364 (6436), 184–188. 10.1126/science.aar2038 30846611 PMC6541389

[B9] Hoang P. Kowalczewski A. Sun S. Winston T. S. Archilla A. M. Lemus S. M. (2021). Engineering Spatial-Organized Cardiac Organoids for Developmental Toxicity Testing. Stem Cell Rep. 16 (5), 1228–1244. 10.1016/j.stemcr.2021.03.013 PMC8185451 33891865

[B10] Hofbauer P. Jahnel S. M. Papai N. Giesshammer M. Deyett A. Schmidt C. (2021). Cardioids Reveal Self-Organizing Principles of Human Cardiogenesis. Cell 184 (12), 3299–3317. 10.1016/j.cell.2021.04.034 34019794

[B11] Hu H. Gehart H. Artegiani B. LÖpez-Iglesias C. Dekkers F. Basak O. (2018). Long-Term Expansion of Functional Mouse and Human Hepatocytes as 3D Organoids. Cell 175 (6), 1591–1606. 10.1016/j.cell.2018.11.013 30500538

[B12] Italia L. Tomasoni D. Bisegna S. Pancaldi E. Stretti L. Adamo M. (2021). COVID-19 and Heart Failure: From Epidemiology during the Pandemic to Myocardial Injury, Myocarditis, and Heart Failure Sequelae. Front. Cardiovasc. Med. 8, 713560. 10.3389/fcvm.2021.713560 34447795 PMC8382715

[B13] Kim H. Kamm R. D. Vunjak-Novakovic G. Wu J. C. (2022). Progress in Multicellular Human Cardiac Organoids for Clinical Applications. Cell Stem Cell 29 (4), 503–514. 10.1016/j.stem.2022.03.012 35395186 PMC9352318

[B14] Kussauer S. David R. Lemcke H. (2019). hiPSCs Derived Cardiac Cells for Drug and Toxicity Screening and Disease Modeling: What Micro- Electrode-Array Analyses Can Tell Us. Cells 8 (11), 1331. 10.3390/cells8111331 PMC6912416 31661896

[B15] Lam N. T. Sadek H. A. (2018). Neonatal Heart Regeneration: Comprehensive Literature Review. Circulation 138 (4), 412–423. 10.1161/circulationaha.118.033648 30571359 PMC6673675

[B16] Lancaster M. A. Renner M. Martin C.-A. Wenzel D. Bicknell L. S. Hurles M. E. (2013). Cerebral Organoids Model Human Brain Development and Microcephaly. Nature 501 (7467), 373–379. 10.1038/nature12517 23995685 PMC3817409

[B17] Lauridsen M. D. Rorth R. Butt J. H. Kristensen S. L. Schmidt M. Moller J. E. (2021). Five-year Risk of Heart Failure and Death Following Myocardial Infarction with Cardiogenic Shock: A Nationwide Cohort Study. Eur. Heart J. Acute Cardiovasc. Care 10 (1), 40–49. 10.1093/ehjacc/zuaa022 33721017

[B18] Lewis-Israeli Y. R. Volmert B. D. Gabalski M. A. Huang A. R. Aguirre A. (2021). Generating Self-Assembling Human Heart Organoids Derived from Pluripotent Stem Cells. J. Vis. Exp. (175), 10.3791/63097. 10.3791/63097 PMC8496916 34605811

[B19] Lewis-Israeli Y. R. Wasserman A. H. Gabalski M. A. Volmert B. D. Ming Y. Ball K. A. (2021). Self-assembling Human Heart Organoids for the Modeling of Cardiac Development and Congenital Heart Disease. Nat. Commun. 12 (1), 5142. 10.1038/s41467-021-25329-5 34446706 PMC8390749

[B20] Lombardi R. Chen S. N. (2020). Editorial of Special Issue “Genetics and Molecular Pathogenesis of Non-ischemic Cardiomyopathies”. Int. J. Mol. Sci. 21 (24), 9398. 10.3390/ijms21249398 PMC7763679 33321793

[B21] Long C. Li H. Tiburcy M. Rodriguez-Caycedo C. Kyrychenko V. Zhou H. (2018). Correction of Diverse Muscular Dystrophy Mutations in Human Engineered Heart Muscle by Single-Site Genome Editing. Sci. Adv. 4 (1), eaap9004. 10.1126/sciadv.aap9004 29404407 PMC5796795

[B22] McElhinney D. B. Geiger E. Blinder J. Woodrow Benson D. Goldmuntz E. (2003). NKX2.5mutations in Patients with Congenital Heart Disease. J. Am. Coll. Cardiol. 42 (9), 1650–1655. 10.1016/j.jacc.2003.05.004 14607454

[B23] Mills R. J. Parker B. L. Quaife-Ryan G. A. Voges H. K. Needham E. J. Bornot A. (2019). Drug Screening in Human PSC-Cardiac Organoids Identifies Pro-proliferative Compounds Acting via the Mevalonate Pathway. Cell Stem Cell 24 (6), 895–907. 10.1016/j.stem.2019.03.009 30930147

[B24] Mills R. J. Humphrey S. J. Fortuna P. R. J. Lor M. Foster S. R. Quaife-Ryan G. A. (2021). BET Inhibition Blocks Inflammation-Induced Cardiac Dysfunction and SARS-CoV-2 Infection. Cell 184 (8), 2167–2182. 10.1016/j.cell.2021.03.026 33811809 PMC7962543

[B25] Miyagawa S. Kainuma S. Kawamura T. Suzuki K. Ito Y. Iseoka H. (2022). Transplantation of IPSC-Derived Cardiomyocyte Patches for Ischemic Cardiomyopathy. medRxiv. Preprint. 10.1101/2021.12.27.21268295 PMC9426776 36051285

[B26] Mokry L. E. Ahmad O. Forgetta V. Thanassoulis G. Richards J. B. (2015). Mendelian Randomisation Applied to Drug Development in Cardiovascular Disease: A Review. J. Med. Genet. 52 (2), 71–79. 10.1136/jmedgenet-2014-102438 25515070

[B27] Pauliks L. B. (2015). The Effect of Pregestational Diabetes on Fetal Heart Function. Expert Rev. Cardiovasc. Ther. 13 (1), 67–74. 10.1586/14779072.2015.988141 25431859

[B28] Richards D. J. Li Y. Kerr C. M. Yao J. Beeson G. C. Coyle R. C. (2020). Human Cardiac Organoids for the Modelling of Myocardial Infarction and Drug Cardiotoxicity. Nat. Biomed. Eng. 4 (4), 446–462. 10.1038/s41551-020-0539-4 32284552 PMC7422941

[B29] Seferovic P. M. Polovina M. M. Coats A. J. S. (2019). Heart Failure in Dilated Non-ischaemic Cardiomyopathy. Eur. Heart J. Suppl. 21 (Suppl. M), M40–M43. 10.1093/eurheartj/suz212 31908615 PMC6937506

[B30] Shanbhag S. M. Greve A. M. Aspelund T. Schelbert E. B. Cao J. J. Danielsen R. (2019). Prevalence and Prognosis of Ischaemic and Non-ischaemic Myocardial Fibrosis in Older Adults. Eur. Heart J. 40 (6), 529–538. 10.1093/eurheartj/ehy713 30445559 PMC6657269

[B31] Shao C. Wang J. Tian J. Tang Y.-d. (2020). Coronary Artery Disease: From Mechanism to Clinical Practice. Adv. Exp. Med. Biol. 1177, 1–36. 10.1007/978-981-15-2517-9_1 32246442

[B32] Shen H. Darehzereshki A. Sucov H. M. Lien C.-L. (2021). Apical Resection and Cryoinjury of Neonatal Mouse Heart. Methods Mol. Biol. 2158, 23–32. 10.1007/978-1-0716-0668-1_2 32857362

[B33] Tadano K. Miyagawa S. Takeda M. Tsukamoto Y. Kazusa K. Takamatsu K. (2021). Cardiotoxicity Assessment Using 3D Vascularized Cardiac Tissue Consisting of Human iPSC-Derived Cardiomyocytes and Fibroblasts. Mol. Ther. Methods Clin. Dev. 22, 338–349. 10.1016/j.omtm.2021.05.007 34514026 PMC8408525

[B34] Takasato M. Er P. X. Chiu H. S. Maier B. Baillie G. J. Ferguson C. (2015). Kidney Organoids from Human iPS Cells Contain Multiple Lineages and Model Human Nephrogenesis. Nature 526 (7574), 564–568. 10.1038/nature15695 26444236

[B35] Tiburcy M. Hudson J. E. Balfanz P. Schlick S. Meyer T. Chang Liao M.-L. (2017). Defined Engineered Human Myocardium with Advanced Maturation for Applications in Heart Failure Modeling and Repair. Circulation 135 (19), 1832–1847. 10.1161/circulationaha.116.024145 28167635 PMC5501412

[B36] Varzideh F. Pahlavan S. Ansari H. Halvaei M. Kostin S. Feiz M.-S. (2019). Human Cardiomyocytes Undergo Enhanced Maturation in Embryonic Stem Cell-Derived Organoid Transplants. Biomaterials 192, 537–550. 10.1016/j.biomaterials.2018.11.033 30529872

[B37] Voges H. K. Mills R. J. Elliott D. A. Parton R. G. Porrello E. R. Hudson J. E. (2017). Development of a Human Cardiac Organoid Injury Model Reveals Innate Regenerative Potential. Development 144 (6), 1118–1127. 10.1242/dev.143966 28174241

[B38] Watson C. L. Mahe M. M. Múnera J. Howell J. C. Sundaram N. Poling H. M. (2014). An *In Vivo* Model of Human Small Intestine Using Pluripotent Stem Cells. Nat. Med. 20 (11), 1310–1314. 10.1038/nm.3737 25326803 PMC4408376

[B39] Wu A. H. (2007). Management of Patients with Non-ischaemic Cardiomyopathy. Heart 93 (3), 403–408. 10.1136/hrt.2005.085761 17322525 PMC1861466

[B40] Xu H. Jiao Y. Qin S. Zhao W. Chu Q. Wu K. (2018). Organoid Technology in Disease Modelling, Drug Development, Personalized Treatment and Regeneration Medicine. Exp. Hematol. Oncol. 7, 30. 10.1186/s40164-018-0122-9 30534474 PMC6282260

[B41] Yang K.-C. Breitbart A. De Lange W. J. Hofsteen P. Futakuchi-Tsuchida A. Xu J. (2018). Novel Adult-Onset Systolic Cardiomyopathy Due to MYH7 E848G Mutation in Patient-Derived Induced Pluripotent Stem Cells. JACC Basic Transl. Sci. 3 (6), 728–740. 10.1016/j.jacbts.2018.08.008 30623132 PMC6314962

[B42] Zhang L. Nomura-Kitabayashi A. Sultana N. Cai W. Cai X. Moon A. M. (2014). Mesodermal Nkx2.5 is Necessary and Sufficient for Early Second Heart Field Development. Dev. Biol. 390 (1), 68–79. 10.1016/j.ydbio.2014.02.023 24613616 PMC4461860

